# Genetic Variations in the Human Angiotensin-ConvertingEnzyme 2 and Susceptibility to Coronavirus Disease-19

**DOI:** 10.1155/2023/2593199

**Published:** 2023-11-29

**Authors:** Taravat Talebi, Tannaz Masoumi, Katayoun Heshmatzad, Mahshid Hesami, Majid Maleki, Samira Kalayinia

**Affiliations:** ^1^Rajaie Cardiovascular Medical and Research Center, Iran University of Medical Sciences, Tehran, Iran; ^2^Cardiogenetic Research Center, Rajaie Cardiovascular Medical and Research Center, Iran University of Medical Sciences, Tehran, Iran

## Abstract

**Background:**

Health and economies are both affected by the coronavirus disease-19 (COVID-19) global pandemic. Angiotensin-converting enzyme 2 (*ACE2*) is a polymorphic enzyme that is a part of the renin-angiotensin system, and it plays a crucial role in viral entry. Previous investigations and studies revealed that severe acute respiratory syndrome coronavirus 2 (SARS-CoV-2) and *ACE2* have a considerable association. Recently, *ACE2* variants have been described in human populations in association with cardiovascular and pulmonary conditions. In this study, genetic susceptibility to COVID-19 in different populations was investigated.

**Methods and Results:**

We evaluated the identified variants based on the predictive performance of 5 deleteriousness-scoring methods and the 2015 American College of Medical Genetics and Genomics (ACMG) guidelines. The results indicated 299 variants within the *ACE2* gene. The variants were analyzed by different *in-silico* analysis tools to assess their functional effects. Ultimately, 5 more deleterious variants were found in the *ACE2* gene.

**Conclusions:**

Collecting more information about the variations in binding affinity between SARS-CoV-2 and host-cell receptors due to *ACE2* variants leads to progress in treatment strategies for COVID-19. The evidence accumulated in this study showed that *ACE2* variants in different populations may be associated with the genetic susceptibility, symptoms, and outcome of SARS-CoV-2 infection.

## 1. Introduction

Coronavirus disease-19 (COVID-19) with first emergence in Wuhan, China, in December 2019 [1, 2] is the consequence of infection with a novel coronavirus naming severe acute respiratory syndrome coronavirus 2 (SARS-CoV-2) recognized as the cause of this new infectious respiratory disease. The World Health Organization [3] on March 2, 2020, denoted this infection as a pandemic [4]. Fever, cough, vomiting, diarrhea, and other symptoms are common among patients with COVID-19. Some cases might develop acute respiratory distress syndrome [5], severe pneumonia, multiple organ failure, and even death [6, 7]. The key characteristic laboratory findings include increased C-reactive protein level, aspartate aminotransferase, lymphopenia, and lactate dehydrogenase [8]. Most COVID-19 affected patients manifest mild symptoms or are asymptomatic [9]. Moreover, susceptibility to COVID-19 varies among age groups, with older individuals being more vulnerable than children [10, 11]. Intensive care unit treatment or hospital admission is required in 10–20% of patients affected with severe disease [12]. Older age, high body mass index, the male sex, and underlying comorbidities such as cardiovascular disease, hypertension, obesity, diabetes, and chronic respiratory disease are risk factors for unfavorable outcomes [13].

**Figure 1:**
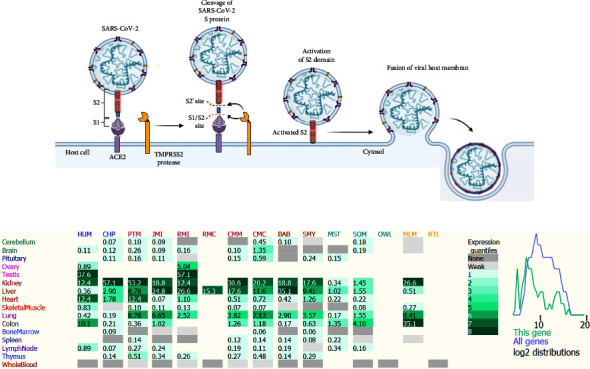
(a) The image illustrates the intracellular interactions between angiotensin-converting enzyme 2 and its ligand severe acute respiratory syndrome coronavirus 2 (SARS-CoV-2). (b) The image shows *ACE2* expression in 15 primates and 16 tissues. The level for significantly expressed genes is color-coded in 8 equally sized bins (light-to-dark green). Light gray is for weak not-accurately measured expression (2 to 8 reads above the intergenic background), while dark gray is for no expression or no sequence conservation (0 read in the gene). The plot on the right shows the distribution of the measured expression values in all tissues for all genes (blue) and for this gene in magic index = log2 (1000 sFPKM). HUM: human, CHP: chimpanzee, PTM: pig-tailed Macaque, JMI: Japanese macaque, RMI: rhesus macaque Indian, RMC: rhesus macaque Chinese, CMM: cynomolgus macaque Mauritian, CMC: cynomolgus macaque Chinese, BAB: olive baboon, SMY: sooty mangabey, MST: common marmoset, SQM: squirrel monkey, OWL: owl monkey, MLM: mouse Lemur, RTL: ring-tailed lemur. This information was obtained from the AceView database (https://www.ncbi.nlm.nih.gov/ieb/research/acembly/).

**Figure 2:**
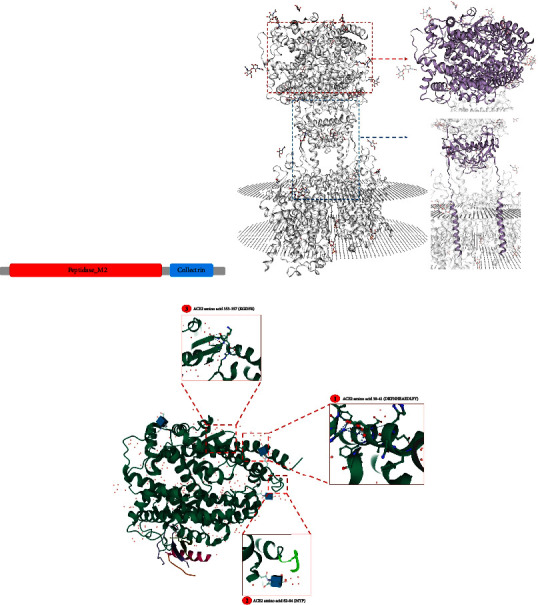
(a) The image depicts the secondary structure of the angiotensin-converting enzyme 2 protein. (b) The image illustrates the dimerization structure of the *ACE2* protein with SWISS-MODEL (https://swissmodel.expasy.org/) ID Q9BYF1. *ACE2* dimerizes via 2 domains: peptidase-M2 and collectrin, which are shown in color. (c) The image demonstrates the crystal structure of *ACE2* with PDB (https://www.rcsb.org/) ID 1R42. The main functional domains of *ACE2* that interact with SARS-CoV-2 are illustrated in the box.

The main host-cell receptor of the spike glycoprotein (S) of SARS-CoV-2 is angiotensin-converting enzyme 2 [14]. This receptor plays a vital role in virus entry into the cell and its infection [15, 16]. Li et al. showed that specific residues in the human *ACE2* (hACE2) receptor are necessary for binding with the pathogen [17]. *ACE2* is an important component of the renin-angiotensin system (RAS) [18, 19], which regulates cardiovascular homeostasis, blood pressure, blood volume, and systemic vascular resistance [20, 21]. *ACE2* is the main enzyme responsible for converting angiotensin II into angiotensin I [1–7]. The imbalance of the RAS caused by the binding of SARS-CoV-2 to *ACE2* is likely to play a role in COVID-19 pathogenesis [22]. Furthermore, *ACE2* is associated with cardiovascular disease, kidney disease, hypertension, stroke, and dyslipidemia [23–26]. In the severe acute respiratory syndrome (SARS) outbreak in 2002–2003, which was caused by SARS-CoV, *ACE2* played the same role as it plays in SARS-CoV-2 infection [27]. The transmembrane protease serine 2 (TMPRSS2) leads to the cleavage of the C-terminal segment of *ACE2* and results in the S protein-driven viral entry [28, 29]. Mutant S proteins can detect host receptors within species [30]. The S protein has 2 subunits: the S1 subunit contains the receptor-binding domain, which targets receptors in the host cells, and the S2 subunit, which regulates membrane fusion between the host cells and the virus [31]. After binding to the *ACE2* receptor, the S protein of SARS-CoV-2 is cleaved by the TMPRSS2 protease at the S1/S2 and S2 sites, leading to the activation of the S2 domain and the membrane fusion of the viral and host membranes (Figure 1(a)) [32]. The abundance of *ACE2* receptors in any organs of the body, including the brain, heart, kidney, nasopharynx, lymph nodes, small intestine, colon, stomach, thymus, skin, spleen, bone marrow, liver, blood vessels, and oral and nasal mucosa, renders them susceptible to infection by SARS-CoV-2 [10, 33]. Previous *in vitro* studies have indicated that there exists a positive robust correlation between SARS-CoV infection and *ACE2* expression [34, 35]. The levels of *ACE2* expression in different tissues are shown in Figure 1(b). *ACE2* is highly expressed in lung alveolar epithelial cells leading to considerable severe lung damage and therefore ARDS acute lung damage and pneumonia as the consequence [36]. The secondary and dimerization structures of the *ACE2* protein are shown in Figures 2(a) and 2(b), respectively. The crystal structure of the *ACE2* receptor is illustrated in Figure 2(c). The binding strength of *ACE2* with SARS-CoV-2 is weaker than that with SARS-CoV, and it is regarded as high as the threshold necessary for the infection of the virus. The S protein is a trimeric glycoprotein expressed in the surface of SARS-CoV-2 virion, which regulates recognition of receptor throughout its membrane fusion and receptor-binding domain [37, 38].

Previous investigations have revealed that the SARS-CoV-2 protein binds to hACE2 through Phe486, Leu455, Ala501, Tyr505, and Gln493. The 31, 41, 82, and 353–357 residues in the *ACE2* receptor are important for its interaction with the S protein of SARS-CoV-2 [17]. Recent clinical studies have demonstrated that male and female patients with COVID-19 exhibit significant differences in incidence and mortality rates. COVID-19 is associated with underlying conditions such as cardiovascular disease and cancer, as well as in specific patients with hypertension consuming antihypertensive medicines [39]. Genetic variations in the *ACE2* gene (Online Mendelian Inheritance in Man (OMIM): 300335) play a critical role in the susceptibility, symptoms, and outcome of SARS-CoV-2 infection in various populations [40]. Some *ACE2* polymorphisms may decrease the association between *ACE2* and the S protein of SARS-CoV [16]. This suggests that an investigation of the functional *ACE2* polymorphisms could promote personalized treatment strategies and precision medicine for COVID-19.

The reported variants of concern (VOCs) included B.1.1.7 (Alpha), B.1.351 (Beta), P.1 (Gamma), B.1.617.2 (Delta), and B.1.1.529 (Omicron) that have mutations in the receptor-binding domain (RBD) and the N-terminal domain (NTD) of the spike protein [41]. These variants lead to increased virulence and transmissibility, reduced neutralization by antibodies, and reduced efficacy of the treatment or vaccination [41]. The development of drugs that target the spike protein is an appropriate therapeutic strategy, which causes an alteration in binding to the *ACE2* receptor [42]. Antiviral drugs, monoclonal antibodies against SARS-CoV-2, anti-inflammatory drugs, and immunomodulatory agents are available as therapeutic strategies [43].

The study aimed to search for the most deleterious variants in the *ACE2* gene associated with COVID-19 and the pathogenesis of the identified variants has been evaluated in silico. We highlighted that the *ACE2* gene variants could guide personalized treatments. *ACE2* polymorphisms could associate with various genetic susceptibility to COVID-19 and treatment outcomes in different ethnic groups. The limitations of this study included that the genomic data in general populations have been examined and the identified *ACE2* variants need to be evaluated in a case-control study. Also, further studies should be done in the future to evaluate the impact of these variants.

## 2. Materials and Methods

### 2.1. Search Strategy and Data Extraction

In the present study, genetic susceptibility to COVID-19 was investigated by evaluating the variants of the *ACE2* gene. The inclusion criteria for variants selection was the variants of *ACE2* which are related to COVID-19.

The combination of the following keywords *ACE2* and COVID-19, *ACE2* variants, and *ACE2* [title/abstract] was used in searching PubMed and Google Scholar. Totally, 64 articles were collected, and after duplicate removal, 22 articles remained in which the variants were collected from these related articles. Duplicate publications and studies with overlapping or insufficient data were excluded. The variants were also collected from the Human Gene Mutation Database (HGMD) (https://www.hgmd.cf.ac.uk/ac/index.php) and ClinVar (https://www.ncbi.nlm.nih.gov/clinvar/).

The Exome Aggregation Consortium (ExAC: https://exac.broadinstitute.org), the 1000 Genomes Project (KGP) (https://www.ncbi.nlm.nih.gov/variation/tools/1000genomes/), the Exome Sequencing Project (https://evs.gs.washington.edu/EVS/), the Genome Aggregation Database (gnomAD v3) (https://gnomad.broadinstitute.org/), Iranome (https://www.iranome.ir/), and the Greater Middle East (GME) Variome Project (https://igm.ucsd.edu/gme/) were used to obtain variants' frequency.

### 2.2. Variants Evaluation

It seems that most of the *ACE2* variants have not been functionally characterized. We evaluated the identified variants based on the 5 prediction tools score according to the threshold value, including Combined Annotation Dependent Depletion (CADD) (https://cadd.gs.washington.edu/home) [44], Sorting Intolerant from Tolerant (SIFT) (https://sift.bii.a-star.edu.sg/) [45], Polymorphism Phenotyping v2 (PolyPhen-2) (https://genetics.bwh.harvard.edu/pph2/) [46], Protein Variation Effect Analyzer (PROVEAN) (https://provean.jcvi.org/index.php) [47], and Mutation Taster (https://www.mutationtaster.org/) [48]. CADD is the most important prediction tool among all bioinformatics software that was used in our manuscript, and the highest CADD Phred for variants evaluation was considered (Phred ≤ 20). Other prediction tools (SIFT, PolyPhen-2, PROVEAN, and MutationTaster) just were explained as descriptive in the range (SIFT: score ≤ 0.05: deleterious, score > 0.05: tolerable; Polyphen-2: score = 0–0.15: benign, score = 0.15–0.85: possibly damaging, score = 0.85–1: probably damaging; PROVEAN: score ≤ −2.5: deleterious, score > −2.5: neutral). We found the variants in the *ACE2* genes that have strong criteria for pathogenesis, i.e., described as a pathogen variant in at least 3 tools. Nomenclature for variants was also confirmed according to the recommendations of the Human Genetic Variation Society (HGVS) (https://varnomen.hgvs.org/). We found the potentially deleterious variants in the *ACE2* gene based on the 2015 American College of Medical Genetics and Genomics (ACMG) guidelines for the interpretation of sequence variants [5].

## 3. Results

### 3.1. Genetic Analysis of hACE2

**Figure 3:**
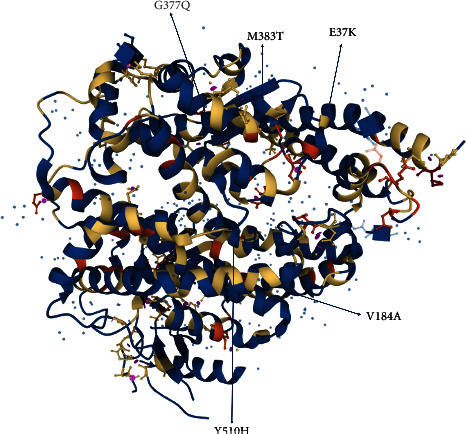
The most pathogenic variants of the *ACE2* gene are displayed by arrows.

The variations in the *ACE2* gene are probably important not only in modulating the host susceptibility to SARS-CoV-2 infection but also in determining the severity of local and systemic tissue damage [49]. In the present study, we collected variant datasets from 6 databases: ExAC, 1KGP, ESP6500, gnomAD, Iranome, and GME. Given that any frequency databases which were used in our study are due to global standards and their population study and methods were different, the minor allele frequency (MAF) of any databases is different. Indeed, we used this information to identify variants with MAF below some specified threshold, which likely relate to disease. ExAC has collected, harmonized, and released exome sequence data from 60706 individuals. 1000G is about common genetic variants with frequencies of at least 1% in the populations studied. ESP6500 is a database of genes and mechanisms that contribute to blood, lung, and heart disorders through NGS data in various populations. gnomAD is a coalition of investigators seeking to aggregate and harmonize exome and genome sequencing data from a variety of large-scale sequencing projects and to make summary data available for the wider scientific community. Iranome is a catalog of genomic variations in the Iranian population. GME generated a coding base reference for the countries found in the Greater Middle East. As we know, the genetic variations of each population are different from the other. Our results revealed 299 variants in the *ACE2* gene. A list of the identified variants in the *ACE2* gene is summarized in Table 1. The majority of the *ACE2* gene variants have yet to be identified functionally. To obtain information about the possibility of the deleterious effects of the identified variants, we evaluated the variants using the *in-silico* prediction of their functional effects. Ultimately, we identified the most deleterious variants in the *ACE2* gene based on prediction tools (Figure 3, Table 2).

### 3.2. Variants of the *ACE2* Gene

Cao et al. explored the allele frequency distribution of 1700 *ACE2* gene variants using China Metabolic Analytics and 1K1000 Genomes [50]. Twenty-five variants located within the *ACE2* gene were collected and cataloged in the Leiden Open Variation Database [14]. Single-nucleotide variations (SNVs) with a low allele frequency appear to be more deleterious than SNVs with a high allele frequency according to some scoring methods [51]. According to a study by Hou et al., 39% and 54% of deleterious variants in the *ACE2* gene are carried by African/African-American and Non-Finnish European populations, respectively. Specifically, 2–10% of deleterious variants in this gene occur in Latino/Admixed American, East Asian, Finnish, and South Asian populations, while Amish and Ashkenazi Jewish populations do not carry deleterious variants in the *ACE2* coding regions [40]. The variants p.Met383Thr, p.Asp427Tyr, and p.Arg514Gly are carried by African/African-American populations, with an allele frequency of 0.003%, 0.01%, and 0.003%, respectively. Additionally, the p.Pro389His variant, with an allele frequency of 0.015%, is carried by Latino/Admixed American populations only [40]. According to a previous study, several *ACE2* variants and alterations in amino acid residues in *ACE2* could affect the association between the *ACE2* receptor and the S protein in SARS-CoV, leading to the conversion of *ACE2* into an efficient/inefficient receptor [17]. Fujikura and Uesaka identified 8 SNVs—namely p.Ser19Pro, p.Thr27Ala, p.Glu35Lys, p.Glu35Asp, p.Glu37Lys, p.Met82Ile, p.Glu329Gly, and p.Asp355Asn—in the *ACE2* gene in the direct contact residues of the S protein of SARS-CoV/SARS-CoV-2 and hACE2 [51]. Residues Arg708/710/716, located in the dimeric interface of the *ACE2* receptor, are a vital component for cleavage by TMPRSS2. This process is required to strengthen the entry of the virus into the host cells [29]. Notably, the variants p.Arg708Trp, p.Arg710Cys, p. Arg710His, and p.Arg716Cys with an allele frequency of 0.01∼0.006% are carried by European populations. East Asian and Latino/Admixed American populations only carry the variants p.Arg708Trp and p.Arg710His, which have an allele frequency of 0.04% and 0.01%, respectively [40]. Several variants, including p.Met383Thr, p.Pro389His, and p.Asp427Tyr, inhibited the interaction between the *ACE2* receptor and the S protein of SARS-CoV-1 in the SARS outbreak in 2002 [17]. There are natural *ACE2* variants that alter the interaction between the virus and the host cells and, as a result, potentially change the susceptibility of the host. In particular, 9 variants—namely, I21V, Q102P, S19P, K26R, E23K, T27A, T92I, N64K, and H378R—were found in the *hACE2* gene, which increased viral binding susceptibility, while 17 variants—namely, K31R, N33I, H34R, E35K, E37K, D38V, Y50F, N51S, M62V, K68E, F72V, Y83H, G326E, G352V, D355N, Q388L, and D509Y—were predicted to decrease the binding affinity of the S protein of SARS-CoV-2 and were, thus, considered protective variants [52]. The variants rs73635825 and rs143936283 present a relatively low binding affinity for the S protein of SARS-CoV-2, which may be associated with potential resistance to infection [49]. Information regarding these variants is not available in Iranome. Three variants—namely, p.Lys26Arg, p.Gly211Arg, and p.Asn720Asp—were more frequently expressed in the Italian population than in the Eastern Asian population. These variants are close to the sequence essential for the binding of the S protein of SARS-CoV-2. The presence of these variants may explain the high mortality rate in Italy compared with China [49, 53]. *ACE2* gene mutation naming Leu584Ala facilitates the SARS-CoV entry into target cells [54]. Cao et al. characterized 32 variants in the *ACE2* gene, among which there were 7 hotspot variants—namely, Lys26Arg, Ile486Val, Ala627Val, Asn638Ser, Ser692Pro, Asn720Asp, and Leu731Ile/Phe—in different populations [50]. Benetti et al. concluded that 3 more common missense variants—namely, p.Gly211Arg, p.Lys26Arg, and p.Asn720Asp—could interfere with both protein structure and its stabilization. Furthermore, the two rare variants of p.Pro389His and p.Leu351Val were predicted to interfere with the binding of the SARS-CoV-2 S protein [4]. Based on the findings of the present study, differential variants in the *ACE2* gene may clarify various susceptibility and outcomes in different ethnic groups.

## 4. Discussion

The *ACE2* receptor acts as an entry point for the coronavirus [55]. In addition to the strategy of using viral replication inhibitors, another strategy in the treatment option is to block the cellular target of the virus, *ACE2* [56]. Certain genomic variants within the *ACE2* gene that modulate its function or expression cause variable susceptibility to SARS-CoV-2 infection [20]. Given the possible connection between circulating *ACE2* levels and COVID-19 severity, recombinant *ACE2* may be a promising treatment option [57]. As a result, tissue-specific *ACE2* expression or plasma *ACE2* levels are considered 2 important factors in the severity of COVID-19. The effects of antihypertensive therapy by both angiotensin-converting enzyme inhibitors (ACE-I) and angiotensin receptor blockers (ARBs) may lead to increased expression levels of *ACE2*. Studies have shown that the increased level of soluble *ACE2* may act as a competitor to SARS-CoV-2 and may, thus, reduce viral penetration into cells and lung tissue [58, 59]. According to a meta-analysis, ACE-I/ARBs reduced the risk of pneumonia and its mortality [60]. The rs2285666 polymorphism may be a predisposing factor for the comorbidities observed in patients with COVID-19 [61, 62]. The population-based frequency of this single-nucleotide polymorphism (SNP) is significantly higher among the Indian population (∼0.6) than among Europeans (0.2) and East Asians (0.55) [21, 50, 62]. In our study, among the Iranian population, we identified a frequency of 0.2575 for this SNP. The results of another study conducted by Srivastava et al. indicated that the frequency of a synonymous coding region variant, rs35803318, was high among Americans (0.15), followed by Europeans (0.055), Caucasians (0.051), and Central Asians (0.021). In the current study, we also detected a frequency of 0.0325 for this SNP among the Iranian population. It appears that some of the identified variants or the cumulative effect of a few of them cause different susceptibility to the entry of viral cells and have a significant effect on the onset and progression of the disease. Therefore, systematic identification of the genetic determinants of COVID-19 susceptibility and the clinical outcome could further explain the current epidemiologic observations, disease pathophysiology, different susceptibilities, and disease severities in different ethnic groups.

In the present study, we conduct a comprehensive systematic investigation on genetic variations in the human genes associated with the coronavirus. The reason for choosing the *ACE2* gene in this study was that variants of this gene may be able to modulate intermolecular interactions with the S protein of SARS-CoV-2 and are associated with altering virulence, pathogenicity, clinical outcome, and COVID-19 susceptibility. In the present study, we provided the dataset of *ACE2* variants (Table 1). The *ACE2* gene variants may be associated with COVID-19 genetic susceptibility which could guide more personalized and individualized treatments for the COVID-19 pandemic [40]. Since *ACE2* gene variants may cause different responses to COVID-19 treatments concerning the components of the RAS system, we recommend case-control studies to investigate the effects of these variants on treatment outcomes. In addition, the testing of the *ACE2* gene polymorphisms has been recommended for patients with COVID-19 undergoing clinical trials with ACE-I/ARBs [9]. Worldwide study on the genes linked to life-threatening instances is required despite the development of many licensed vaccinations, the mutation of coronaviruses, and the potential for pandemics. It is also necessary to obtain information on variants for population-appropriate vaccines against SARS-CoV-2 infection.

This study aimed to search for the most deleterious variants associated with COVID-19, and the pathogenesis of the identified variants has been investigated *in silico*. We selected the variants with the highest CADD score and were considered as deleterious, damaging, and disease causing in at least three prediction tools. Also, the MAF of the selected variants in the frequency databases was very low, and these variants can be very important in the incidence of the disease (Figure 3, Table 2). Finally, we found the five variants caused the changes in amino acid residues of the extracellular domain of the *ACE2* receptor (residues 18–740) that includes a zinc-binding site (residues 374–378, His-Glu-Met-Gly-His). The mutated residues are located in the extracellular domain which plays an important role in the main activity of the *ACE2* protein, and these variants can consequently disturb its normal function. The S protein of SARS-CoV-2 is identified by the extracellular peptidase domain of the *ACE2* receptor and leads to the binding of the virus to the host cell. Probably, each of these five deleterious variants mentioned in this study caused a disturbance in the structure of the *ACE2* receptor, which may be effective in the incidence of this disease. The c.1129G > T variant in the *ACE2* gene caused the Gly377Gln substitution within the extracellular domain of the receptor. This residue is located in the zinc-binding site (positions 374–378) that is involved in binding. The E37K variant is in the direct contact residues of hACE2 and the S protein that play a role in the entry of the virus into the host cells. The initial attachment of the S protein to the receptor has caused the exposure of the most important amino acids for binding (residues 22–57). The main functional domains of the *ACE2* receptor that interact with SARS-CoV-2 are illustrated in Figure 2(c). The c.109G > A variant in the *ACE2* gene caused the Glu37Lys substitution within the main functional domains of *ACE2* (residues 30–41). Also, amino acid glycine at position 37 is the main residue at the interface.

According to this study, the five deleterious variants in the *ACE2* gene may clarify various susceptibility and outcomes in different ethnic groups. These *ACE2* variants and alterations in amino acid residues in the receptor alter the interaction between the virus and host cells, resulting in altering the host susceptibility. Therefore, we recommend further research to identify the effect of the most pathogenic variants on the binding affinity. Also, the identified pathogenic variants in the *ACE2* gene may affect the clinical efficacy of drugs for COVID-19, which is better investigated. We suggest that the frequency of these deleterious variants in different populations is investigated in the future so that the necessary preparations for the disease are considered in populations carrying these variants.

The tissue-specific *ACE2* expression and plasma *ACE2* levels, and density of *ACE2* receptors are key factors of the difference in the severity and incidence of the disease in various countries. Also, the levels of *ACE2* expression vary in different populations and various human tissues (Figure 1(b)). SNPs affect gene expression and lead to a change in the outcome of the disease. We recommend that these factors be investigated in individuals with these variants in different populations that could promote personalized treatment strategies and precision medicine for COVID-19. Such studies may affect accurate medical interventions and the design of specific diagnostic and therapeutic methods for coronavirus. The present study can be useful for better understanding interindividual clinical variability, and the severity and susceptibility of this disease in different ethnic groups.

The mechanisms resulting from the functional foods-based treatments included the reduced expression of *ACE2* receptors in cells, inhibiting necessary enzymes in SARS-CoV-2, and decreased proinflammatory cytokines that can help the body fight during illness [63]. The mentioned variants that modulate the *ACE2* function and expression cause variable susceptibility to SARS-CoV-2 infections. It seems to be beneficial for patients carrying these variants to use the functional foods-based treatments that lead to the reduced expression of *ACE2* receptors in the cells. Therefore, we recommend further research to identify the effect of the most pathogenic variants in different populations on the *ACE2* tissue expression, plasma *ACE2* levels, and binding affinity, leading to improved therapeutic strategies and precision medicine for COVID-19. We suggested that the testing of the polymorphisms and the most pathogenic variants in the *ACE2* gene should be considered when determining the type of drugs in patients with more severe symptoms. According to the studies, numerous polymorphisms are associated with high *ACE2* tissue expression and higher severity, whereas some polymorphisms are associated with low *ACE2* tissue expression and lesser severity. As a result, the treatment outcomes in COVID-19 patients are influenced by the *ACE2* variants. The spike protein mutations increased the viral attachment and subsequent entry into host cells. The structural target for available drugs and treatments is the high binding affinity of the spike protein and the receptor. It appears that some of the identified variants and their cumulative effects of them cause different susceptibility to the entry of viral cells and have a significant effect on the used therapeutics and vaccination effectiveness. Given the possibility that treatment-resistant variants may emerge that could lead to destructive and irrecoverable impacts on global health, continuous viral surveillance of new variants should be performed using viral genomic sequencing. Both the virus and receptor variants are two important factors in the susceptibility and severity of this disease. Therefore, we suggest that both factors should be considered to select the proper therapeutic strategy. Despite the production of several approved vaccines, mass vaccination, recommending vaccine boosters, the latest novel therapeutics available, and food-based treatments, the significant progress made so far in stopping the spread of SARS-CoV-2 is threatened by the continued emergence of new variant strains of SARS-CoV-2. It also highlights further investigation on genes associated with life-threatening cases is necessary due to adaptive mutations in the viral genome that can change the pathogenic potential of this virus. The evaluation of pathogenic variants in the *ACE2* gene in male and female genders and different populations with the appropriate therapeutic strategies can be effective to prevent infections among populations at risk of SARS-CoV-2 infections resulting from possible viral variants.

## 5. Conclusions

The detection of SNP genotypes is urgently needed to discover likely genetic risk factors for severe outcomes. The identification of variants may have a significant impact on the variability of the COVID-19 course and may confer precision medicine interventions, treatment individualization and design, and inexpensive and accurate DNA-based tests for the coronavirus. Our genetic analysis of variants in the *hACE2* gene suggests that the *ACE2* variants may be associated with COVID-19 susceptibility and clinical outcomes.

## Figures and Tables

**Table 1:** Genetic variations in *ACE2* gene (NM_021804.2).

Position on chromosome X	Nucleotide change	Amino acid change	dbSNP	CADD Phred	SIFT	Polyphen2	PROVEAN	Mutation taster	ExAc	1000 genomes project	ESP6500	gnomAD	Iranome	GME
15580015	c.^*∗*^13T > G	NA	rs370013094	5.28	NA	NA	NA	P	0.00001	NA	0.0095	NA	NA	NA
15580093	c.2353G > A	D785N	rs373153165	22.7	DE	Benign	NE	P	0.00003	NA	0.0095	NA	NA	NA
15580101	c.2345C > T	A782V	rs147487891	8.04	DE	Benign	NE	P	0.00005	NA	0.0095	NA	NA	NA
15580115	c.2331A > G	G777=	rs375252585	10.37	NA	NA	NA	DC	0.00009	NA	0.0095	0.00014	NA	NA
15580185	c.2310 − 49G > A	NA	rs369519219	1.28	NA	NA	NA	NA	0.00001	NA	0.0095	NA	NA	NA
15582154	c.2302C > T	R768W	rs140016715	6.86	DE	PRD	DE	DC	0.00001	NA	0.0095	NA	NA	NA
15582235	c.2221A > G	I741V	rs372923812	10.41	DE	Benign	NE	P	0.00009	NA	0.0095	0.00009	NA	0.00137
15582265	c.2191C > T	L731F	rs147311723	22.2	DE	PRD	NE	DC	0.00174	0.0048	0.6438	0.00360	NA	NA
15582270	c.2186C > T	P729L	rs375923132	23.4	DE	Benign	DE	DC	0.00001	NA	0.0095	0.00005	NA	NA
15582280	c.2176G > C	G726R	rs139980377	25.5	DE	PRD	DE	DC	0.00002	NA	0.0095	NA	NA	0.00068
15582298	c.2158A > G	N720D	rs41303171	22.1	DE	Benign	NE	P	0.01654	0.0045	1.7514	0.01477	0.00625	0.00137
15582310	c.2146C > T	R716C	rs144869363	3.58	DE	PRD	NE	P	NA	NA	0.0095	NA	NA	NA
15582327	c.2129G > A	R710H	rs370187012	26.1	DE	PRD	NE	DC	0.00004	NA	0.0095	0.00005	NA	NA
15582338	c.2118G > A	M706I	rs372986872	13.00	DE	Benign	NE	P	0.00001	NA	0.0095	NA	NA	NA
15584353	c.2114 + 23G > A	NA	rs376962756	1.82	NA	NA	NA	P	0.00001	NA	0.0095	NA	NA	NA
15584372	c.2114 + 4A > G	NA	rs371381538	18.2	NA	NA	NA	DC	0.00001	NA	0.0095	NA	NA	NA
15584416	c.2074T > C	S692P	rs149039346	7.76	DE	POD	NE	P	0.00043	0.00210	0.1799	0.00210	NA	NA
15585806	c.1997 + 43T > G	NA	rs374566217	4.06	NA	NA	NA	P	0.00004	NA	0.0095	0.00005	NA	NA
15585826	c.1997 + 23C > G	NA	rs368398312	4.891	NA	NA	NA	P	0.00001	NA	0.0095	NA	NA	NA
15588426	c.1888G > C	D630H	rs140312271	20.8	DE	POD	NE	P	0.00001	NA	0.0095	NA	NA	NA
15588474	c.1840G > T	A614S	rs201715513	5.18	DE	Benign	NE	P	0.00022	NA	0.0284	0.00009	NA	NA
5589701	c.1837 + 46T > G	NA	rs367888640	4.08	NA	NA	NA	P	0.00002	NA	0.0095	0.00005	NA	NA
15589702	c.1837 + 45C > T	NA	rs4646169	0.83	NA	NA	NA	P	0.00183	0.0048	0.6343	0.00368	NA	NA
15589725	c.1837 + 22G > C	NA	rs4646168	1.46	NA	NA	NA	P	NA	0.0318	4.3927	0.03254	0.00062	NA
15589729	c.1837 + 18A > G	NA	rs368695026	5.46	NA	NA	NA	P	0.00002	NA	0.0189	NA	NA	NA
15589738	c.1837 + 8del1	NA	NA	NA	NA	NA	NA	NA	NA	NA	0.2548	NA	NA	NA
15589740	c.1837 + 3_1837 + 6del4	NA	NA	NA	NA	NA	NA	NA	NA	NA	0.0189	NA	NA	NA
15589793	c.1791C > A	D597E	rs145437639	1.36	DE	Benign	NE	P	0.00006	NA	0.0284	0.00050	NA	NA
15589801	c.1783C > G	L595V	rs148036434	25.1	DE	PRD	DE	DC	0.00002	NA	0.0189	NA	NA	NA
15589806	c.1778C > A	T593N	rs140857723	14.42	DE	Benign	NE	P	0.00001	NA	0.0095	0.00005	NA	NA
15589838	c.1746G > T	R582S	rs372924787	0.48	DE	Benign	NE	P	0.00001	NA	0.0095	0.00009	NA	NA
15589839	c.1745G > A	R582K	rs150172355	1.29	DE	Benign	NE	P	0.00003	NA	0.0095	NA	NA	NA
15589896	c.1688C > T	S563L	rs375352455	37	DE	PRD	DE	DC	NA	NA	0.0095	NA	NA	NA
15589925	c.1665 − 7del1	NA	NA	NA	NA	NA	NA	NA	NA	NA	0.0686	NA	NA	NA
15589941	c.1665 − 23del1	NA	NA	NA	NA	NA	NA	NA	NA	NA	0.0098	NA	NA	NA
15590297	c.1664 + 27C > G	NA	rs369635645	3.68	NA	NA	NA	NA	NA	NA	0.0095	0.00005	NA	NA
15590348	c.1640C > G	S547C	rs373025684	24.2	DE	PRD	DE	DC	NA	NA	0.0095	0.00009	NA	NA
15590473	c.1542 − 27T > G	NA	rs374990263	3.36	NA	NA	NA	P	NA	NA	0.0189	0.00005	NA	NA
15590479	c.1542 − 33G > A	NA	rs369311079	0.27	NA	NA	NA	P	NA	NA	0.0095	NA	NA	NA
15590482	c.1542 − 36C > G	NA	rs372629764	0.25	NA	NA	NA	NA	NA	0.0003	0.0284	0.00023	NA	NA
15590489	c.1542 − 43G > A	NA	rs377075452	1.00	NA	NA	NA	P	NA	NA	0.0095	NA	NA	NA
15591528	c.1503A > G	A501=	rs368996871	5.32	NA	NA	NA	DC	0.00001	NA	0.0095	NA	NA	NA
15593780	c.1442 + 9A > G	NA	rs374011627	11.19	NA	NA	NA	P	0.00001	NA	0.0095	NA	NA	NA
15593945	c.1298 − 12T > C	NA	rs377717225	27.3	NA	NA	NA	DC	0.00001	NA	0.0189	0.00014	NA	NA
15596175	c.1297 + 37T > C	NA	rs371025504	8.27	NA	NA	NA	P	0.00020	0.0005	0.0095	0.00032	NA	NA
15596186	c.1297 + 26T > A	NA	rs375551860	2.93	NA	NA	NA	NA	0.00001	NA	0.0095	0.00005	NA	NA
15596193	c.1297 + 19G > A	NA	rs377563617	7.25	NA	NA	NA	DC	0.00004	NA	0.0189	NA	NA	NA
15596357	c.1152A > G	A384=	rs138689466	9.86	NA	NA	NA	DC	NA	NA	0.0095	NA	NA	NA
15596376	c.1133A > G	H378R	rs142984500	25.5	DE	PRD	DE	DC	0.00007	NA	0.0189	0.00014	NA	NA
15599359	c.1055G > T	G352V	rs370610075	23.5	DE	PRD	DE	DC	0.00001	NA	0.0095	NA	NA	NA
15599392	c.1022A > G	K341R	rs138390800	17.20	DE	Benign	NE	P	0.00047	0.0008	0.1704	0.00101	NA	0.00069
15599428	c.986A > G	E329G	rs143936283	9.50	DE	Benign	NE	P	0.00002	NA	0.0189	0.00009	NA	NA
15599481	c.933C > G	A311=	rs373974232	5.54	NA	NA	NA	P	NA	NA	0.0095	0.00009	0.001250	NA
15605847	c.802 + 29C > T	NA	rs367784090	6.62	NA	NA	NA	P	0.00006	0.0003	0.0095	0.00009	NA	NA
15605853	c.802 + 23C > T	NA	rs368545997	0.63	NA	NA	NA	P	NA	NA	0.0189	NA	NA	NA
15606009	c.697 − 28T > C	NA	rs375070525	6.54	NA	NA	NA	P	NA	NA	0.0095	NA	NA	NA
15607464	c.696 + 3A > G	NA	rs369559816	19.23	NA	NA	NA	DC	0.00002	NA	0.0095	NA	NA	NA
15607508	c.655C > T	R219C	rs372272603	17.36	DE	PRD	NE	DC	0.00031	0.0003	0.0379	0.00057	NA	NA
15607546	c.617A > G	D206G	rs142443432	18.18	DE	Benign	NE	DC	0.00024	NA	0.0284	0.00039	NA	NA
15607580	c.584 − 5_584 − 2del4	NA	NA	NA	NA	NA	NA	NA	NA	NA	0.0098	NA	NA	NA
15607586	c.584 − 8_584 − 7insA	NA	NA	NA	NA	NA	NA	P	NA	NA	2.6855	NA	NA	NA
15607619	c.584 − 40T > G	NA	rs375208456	11.90	NA	NA	NA	P	0.00007	NA	0.0095	NA	NA	NA
15610303	c.439 + 49G > C	NA	rs369271593	1.55	NA	NA	NA	NA	0.00004	NA	0.0095	0.00331	NA	NA
15610328	c.439 + 24G > A	NA	rs73195520	5.70	NA	NA	NA	P	0.00230	0.0013	0.2653	0.0013	0.00062	NA
15610492	c.346 − 47C > T	NA	rs369762601	0.51	NA	NA	NA	P	0.00001	NA	0.0095	NA	NA	NA
15612922	c.345 + 46T > G	NA	rs374174485	5.24	NA	NA	NA	P	0.00003	NA	0.0095	NA	NA	NA
15612947	c.345 + 21G > A	NA	rs377404656	6.85	NA	NA	NA	P	0.00002	NA	0.0095	NA	NA	NA
15612964	c.345+4C > T	NA	rs370730253	6.32	NA	NA	NA	DC	0.00009	NA	0.0095	NA	NA	NA
15612969	c.344G > A	R115Q	rs201900069	9.32	DE	Benign	NE	P	0.00021	0.0003	0.0095	0.00014	NA	NA
15612993	c.320T > C	V107A	rs139773121	0.52	DE	Benign	NE	P	0.00002	NA	0.0189	NA	NA	NA
15613006	c.307A > C	N103H	rs143158922	11.78	DE	POD	NE	P	0.00001	NA	0.0284	0.00005	NA	NA
15613060	c.253C > T	L85=	rs376392863	5.8	NA	NA	NA	P	0.00003	NA	0.0095	0.00009	NA	NA
15613115	c.198G > A	G66=	rs370473130	1.60	NA	NA	NA	P	0.00002	NA	0.0095	0.00014	NA	NA
15618918	c.117G > A	L39=	rs199804629	0.44	NA	NA	NA	P	0.00019	0.0003	0.0095	0.00005	NA	NA
15618926	c.109G > A	E37K	rs146676783	34	DE	POD	NE	DC	0.00002	NA	0.0284	0.00009	NA	NA
15618933	c.102C > T	H34=	rs368655410	0.01	NA	NA	NA	P	0.00058	NA	0.0189	0.00073	0.00062	0.00068
15618975	c.60C > T	T20=	rs372345059	1.60	NA	NA	NA	P	0.00001	NA	0.0095	NA	NA	NA
15618980	c.55T > C	S19P	rs73635825	8.77	DE	POD	NE	P	0.00034	0.0008	0.142	0.00082	NA	NA
15580089	c.2357T > C	I786T	NA	6.51	DE	POD	NE	P	NA	NA	NA	NA	0.00187	NA
15582209	c.2247G > A	Val749=	rs35803318	4.18	NA	NA	NA	DC	0.03904	0.0209	NA	0.02655	0.03250	0.03196
15582333	c.2123G > A	R708Q	rs769062069	24.9	DE	Benign	NE	DC	0.00001	NA	NA	NA	0.00187	NA
15584288	c.2114 + 88G > A	NA	rs4646180	2.87	NA	NA	NA	P	NA	0.0167	NA	0.01695	0.00064	NA
15584310	c.2114 + 66G > A	NA	NA	1.04	NA	NA	NA	P	NA	NA	NA	NA	0.00125	NA
15584324	c.2114 + 50_2114 + 52delCAA	NA	rs777042582	NA	NA	NA	NA	NA	NA	NA	NA	0.00014	0.00375	NA
15584350	c.2114 + 26T > C	NA	NA	0.03	NA	NA	NA	P	NA	NA	NA	NA	0.00062	NA
15584420	c.2070T > C	Asn690=	rs4646179	7.82	NA	NA	NA	P	0.00713	0.0225	NA	0.01744	0.00437	0.01726
15584701	c.1998 − 209T > C	NA	rs4646178	1.96	NA	NA	NA	P	NA	0.0167	NA	0.01657	0.00065	NA
15584727	c.1998 − 235A > G	NA	rs762461812	1.98	NA	NA	NA	P	NA	0.0003	NA	0.00068	0.00142	NA
15585987	c.1897 − 38G > A	NA	rs61433707	5.95	NA	NA	NA	DC	0.00179	0.0058	NA	0.00423	0.00187	NA
15586132	c.1897 − 183G > A	NA	rs766780343	0.51	NA	NA	NA	P	NA	0.0021	NA	0.00005	0.00194	NA
15588364	c.1896 + 54A > C	NA	NA	3.59	NA	NA	NA	P	NA	NA	NA	NA	0.00062	NA
15588723	c.1838 − 247A > C	NA	NA	1.01	NA	NA	NA	P	NA	NA	NA	NA	0.00265	NA
15589621	c.1837 + 126T > G	NA	rs4646170	4.36	NA	NA	NA	P	NA	0.0270	NA	0.02914	0.00063	NA
15589680	c.1837 + 67C > T	NA	rs142060377	1.50	NA	NA	NA	P	NA	0.0011	NA	0.00041	0.00062	NA
15589978	c.1665 − 59G > A	NA	rs4646167	0.97	NA	NA	NA	P	NA	0.0225	NA	0.01711	0.00476	NA
15590231	c.1664 + 93A > C	NA	rs146750287	4.91	NA	NA	NA	P	NA	0.0016	NA	0.00164	0.00133	NA
15590454	c.1542 − 8C > T	NA	rs767194965	3.42	NA	NA	NA	P	0.00003	NA	NA	NA	0.00125	NA
15590501	c.1542 − 55_1542 − 54insA	NA	NA	NA	NA	NA	NA	P	NA	NA	NA	NA	0.00125	NA
15590547	c.1542 − 102_1542 − 101delTT	NA	NA	NA	NA	NA	NA	P	NA	NA	NA	NA	0.00063	NA
15590562	c.1542 − 116T > A	NA	rs768948617	2.53	NA	NA	NA	P	NA	0.0003	NA	0.00041	0.00854	NA
15591550	c.1481A > T	D494V	rs765152220	31	DE	PRD	DE	DC	0.00007	NA	NA	NA	0.00062	NA
15591578	c.1453G > C	V485L	NA	23.7	DE	PRD	NE	NA	NA	NA	NA	NA	0.00187	NA
15591685	c.1443 − 97delA	NA	rs11340646	NA	NA	NA	NA	NA	NA	0.0016	NA	NA	0.2943	NA
15591685	c.1443 − 98_1443 − 97delAA	NA	rs769765211	NA	NA	NA	NA	NA	NA	NA	NA	NA	0.1374	NA
15591685	c.1443 − 99_1443 − 97delAAA	NA	rs775397699	NA	NA	NA	NA	NA	NA	NA	NA	NA	0.01202	NA
15591685	c.1443 − 98_1443 − 97dupAA	NA	rs11340646	NA	NA	NA	NA	NA	NA	0.0016	NA	NA	NA	NA
15591710	c.1443 − 122C > T	NA	NA	23.6	NA	NA	NA	P	NA	NA	NA	NA	0.00079	NA
15593698	c.1442 + 90_1442 + 91delCA	NA	rs200260858	NA	NA	NA	NA	NA	NA	0.0074	NA	NA	0.007015	NA
15593877	c.1354T > G	F452V	NA	26.3	DE	POD	DE	DC	NA	NA	NA	NA	0.00062	NA
15593752	c.1442 + 37T > G	NA	NA	26.3	NA	NA	NA	DC	NA	NA	NA	NA	0.00062	NA
15596144	c.1297 + 68_1297 + 69insCTTAT	NA	rs4646158	NA	NA	NA	NA	P	NA	0.1658	NA	0.27022	0.6093	NA
15599413	c.1001C > T	T334M	NA	18.52	DE	PRD	NE	P	NA	NA	NA	NA	0.00187	NA
15599422	c.992C > T	S331F	NA	24.7	DE	POD	DE	DC	NA	NA	NA	NA	0.00062	NA
15605852	c.802 + 24G > A	NA	rs4646140	0.60	NA	NA	NA	P	0.02215	0.0601	NA	0.03364	0.01500	NA
15603508	c.900 + 90C > A	NA	rs41297301	5.10	NA	NA	NA	P	NA	0.0037	NA	0.01453	0.01142	NA
15603509	c.900 + 89G > C	NA	NA	0.61	NA	NA	NA	P	NA	NA	NA	NA	0.00133	NA
15603813	c.803 − 118G > A	NA	NA	1.52	NA	NA	NA	P	NA	NA	NA	NA	0.00081	NA
15606091	c.697 − 110A > G	NA	rs755820352	4.58	NA	NA	NA	P	NA	NA	NA	0.00014	0.00094	NA
15607282	c.696 + 185T > A	NA	rs868731794	1.74	NA	NA	NA	P	NA	NA	NA	0.00005	0.00131	NA
15607374	c.696 + 93T > A	NA	NA	0.67	NA	NA	NA	P	NA	NA	NA	NA	0.00062	NA
15607411	c.696 + 56A > C	NA	NA	5.11	NA	NA	NA	P	NA	NA	NA	NA	0.00062	NA
15607489	c.674A > G	D225G	NA	25	DE	PRD	DE	DC	NA	NA	NA	NA	0.00125	NA
15607567	c.596A > G	Y199C	rs750145841	24	DE	PRD	DE	DC	NA	0.00001	NA	NA	0.00125	NA
15607587	c.584 − 8dupA	NA	rs776459296	NA	NA	NA	NA	NA	NA	NA	NA	NA	0.00126	NA
15607650	c.584 − 71A > G	NA	rs971249	2.22	NA	NA	NA	P	NA	0.1968	NA	0.30454	0.5814	NA
15609990	c.440 − 11T > C	NA	NA	15.68	NA	NA	NA	P	NA	NA	NA	NA	0.00062	NA
15610348	c.439 + 4G > A	NA	rs2285666	6.88	NA	NA	NA	P	0.17702	0.3502	NA	0.22879	0.2575	NA
15610506	c.346 − 61A > G	NA	rs4646135	3.30	NA	NA	NA	P	NA	0.0286	NA	0.03102	0.00062	NA
15610588	c.346 − 143A > T	NA	rs73195521	2.73	NA	NA	NA	P	NA	0.0013	NA	0.00303	0.00081	NA
15613138	c.187 − 12C > T	NA	rs757019762	0.74	NA	NA	NA	P	0.00003	NA	NA	NA	0.00125	NA
15618737	c.186 + 112G > A	NA	rs757774161	9.65	NA	NA	NA	P	NA	0.0005	NA	0.00005	0.00127	NA
15618769	c.186 + 80C > A	NA	rs187959864	9.09	NA	NA	NA	P	NA	0.0003	NA	0.00009	0.00690	NA
15618770	c.186 + 79T > A	NA	NA	12.15	NA	NA	NA	P	NA	NA	NA	NA	0.00627	NA
15618774	c.186 + 75G > A	NA	NA	11.13	NA	NA	NA	P	NA	NA	NA	NA	0.00564	NA
15618775	c.186 + 74G > A	NA	NA	7.57	NA	NA	NA	P	NA	NA	NA	NA	0.00626	NA
15618776	c.186 + 73G > A	NA	NA	6.07	NA	NA	NA	P	NA	NA	NA	NA	0.00438	NA
15618828	c.186 + 21T > A	NA	rs748232717	1.60	NA	NA	NA	P	0.00008	NA	NA	NA	0.00062	NA
15618856	c.179A > G	Q60R	rs759162332	22.8	DE	PRD	NE	DC	0.00002	NA	NA	NA	0.00125	NA
15619036	c.−2C > T	NA	rs761675562	13.71	NA	NA	NA	DC	0.00000	NA	NA	0.00005	0.00062	NA
15618958	c.77A > G	K26R	rs4646116	10.75	DE	Benign	NE	P	0.00368	0.0021	NA	0.00315	NA	0.00068
15599363	c.1051C > G	L351V	NA	22.6	DE	PRD	NE	P	NA	NA	NA	NA	NA	NA
15596380	c.1129G > T	G377Q	NA	28.2	DE	PRD	DE	DC	NA	NA	NA	NA	NA	NA
15612712	g.15630835A > G	NA	rs6632680	7.26	NA	NA	NA	P	NA	0.2705	NA	0.41458	NA	NA
15525037	g.15543160A > G	NA	rs4830965	4.04	NA	NA	NA	P	NA	0.1756	NA	0.28718	NA	NA
15621438	g.15639561T > G	NA	rs1548474	2.1	NA	NA	NA	P	NA	0.30406	NA	0.3232	NA	NA
15522176	g.15540299T > C	NA	rs1476524	NA	NA	NA	NA	P	NA	0.3105	NA	0.38494	NA	NA
15618974	c.61A > G	I21V	rs778030746	0.09	DE	Benign	NE	P	0.00002	NA	NA	NA	NA	NA
15618968	c.67G > A	E23K	rs756231991	33	DE	Benign	NE	P	0.00001	NA	NA	NA	NA	NA
15618956	c.79A > G	T27A	rs781255386	12.93	DE	Benign	NE	P	0.00001	NA	NA	NA	NA	NA
15613121	c.192T > A	N64K	rs1199100713	0.0	DE	Benign	NE	P	NA	NA	NA	0.00009	NA	NA
15613008	c.305A > C	Q102P	rs1395878099	17.14	DE	Benign	NE	P	NA	NA	NA	0.00005	NA	NA
15618943	c.92A > G	K31R	NA	11.41	DE	Benign	NE	P	NA	NA	NA	NA	NA	NA
15618937	c.98A > T	N33I	NA	23.6	DE	Benign	DE	DC	NA	NA	NA	NA	NA	NA
15618934	c.101A > G	H34R	NA	0.01	DE	PRD	NE	P	NA	NA	NA	NA	NA	NA
15618932	c.103G > A	E35K	rs1348114695	26.2	DE	Benign	NE	P	NA	NA	NA	NA	NA	NA
15618922	c.113A > T	D38	NA	15.01	DE	NA	NA	DC	NA	NA	NA	NA	NA	NA
15618886	c.149A > T	Y50	rs1192192618	23.4	DE	Benign	NE	DC	NA	NA	NA	NA	NA	NA
15618883	c.152A > G	N51S	rs1569243690	25.2	DE	PRD	DE	DC	NA	NA	NA	NA	NA	NA
15618851	c.184A > G	M62V	rs1325542104	16.31	DE	Benign	NE	DC	NA	NA	NA	NA	NA	NA
15613111	c.202A > G	Y83H	rs755691167	14.16	DE	PRD	DE	P	0.00001	NA	NA	NA	NA	NA
15599437	c.977G > A	G326E	rs759579097	21.2	DE	Benign	NE	P	0.00001	NA	NA	NA	NA	NA
15599351	c.1063G > A	D355N	rs961360700	23.8	DE	PRD	DE	DC	NA	NA	NA	NA	NA	NA
15596346	c.1163A > T	Q388L	rs751572714	19.53	DE	Benign	NE	DC	0.00002	NA	NA	NA	NA	NA
15591506	c.1525G > T	D509Y	NA	25.9	DE	PRD	DE	DC	NA	NA	NA	NA	NA	NA
15596361	c.1148T > C	M383T	rs1396769231	27.7	DE	PRD	DE	DC	NA	NA	NA	NA	NA	NA
15613067	c.246G > A	M82I	Rs766996587	0.01	DE	Benign	NE	P	0.00001	NA	NA	0.00014	NA	NA
15591574	c.1457G > T	G86V	NA	28.9	DE	NA	NA	DC	NA	NA	NA	NA	NA	NA
15588434	c.1880C > T	A627V	rs748163894	25.3	DE	POD	NE	DC	0.00001	NA	NA	NA	NA	NA
15615453	c.187 − 2327T > C	NA	rs4646127	2.8	NA	NA	NA	P	0.1907	NA	NA	0.30725	NA	NA
15617736	c.186 + 1113C > T	NA	rs4646120	0.55	NA	NA	NA	P	0.2646	NA	NA	0.43227	NA	NA
15599893	c.901 − 380_901 − 379insTTAA	NA	rs4646148	NA	NA	NA	NA	NA	NA	0.1717	NA	NA	NA	NA
15614145	c.187 − 1019C > T	NA	rs2023802	0.29	NA	NA	NA	P	NA	0.1958	NA	NA	NA	NA
15616796	c.186 + 2053A > G	NA	rs4646124	9.4	NA	NA	NA	P	NA	0.1963	NA	0.30057	NA	NA
15597043	c.1071 − 605T > G	NA	rs4646156	0.42	NA	NA	NA	P	NA	0.1974	NA	0.30113	NA	NA
15614664	c.187 − 1538dupA	NA	rs397822493	NA	NA	NA	NA	NA	NA	0.1642	NA	0.26729	NA	NA
15608499	c.584 − 920A > T	NA	rs2048683	1.30	NA	NA	NA	P	NA	0.1966	NA	0.30127	NA	NA
15600880	c.901 − 1367dupT	NA	rs11394305	NA	NA	NA	NA	NA	NA	0.1751	NA	NA	NA	NA
15600691	c.901 − 1178G > C	NA	rs2316904	1.9	NA	NA	NA	P	NA	0.1725	NA	0.27328	NA	NA
15600744	c.901 − 1231A > T	NA	rs4646147	4.5	NA	NA	NA	P	NA	0.1719	NA	0.27554	NA	NA
15601274	c.901 − 1761C > A	NA	rs2316903	3.48	NA	NA	NA	P	NA	0.1722	NA	0.27513	NA	NA
15590829	c.583 + 884G > C	NA	rs757066	10.65	NA	NA	NA	P	NA	0.1436	NA	0.24908	NA	NA
15604865	c.802 + 1011C > T	NA	rs1514279	1.35	NA	NA	NA	P	NA	0.1968	NA	0.30189	NA	NA
15597835	c.1071 − 1397G > T	NA	rs4646153	1.34	NA	NA	NA	P	NA	0.1682	NA	0.26963	NA	NA
15598024	c.1070 + 1320T > G	NA	rs4646152	7.2	NA	NA	NA	P	NA	0.1677	NA	0.27064	NA	NA
15600215	c.901 − 702T > G	NA	rs2048684	1.45	NA	NA	NA	P	NA	0.1677	NA	0.27083	NA	NA
15603064	c.900 + 534C > T	NA	rs4646142	9.31	NA	NA	NA	P	0.3587	NA	NA	0.23549	NA	NA
15610349	c.439 + 3dupA	NA	rs756737634	NA	NA	NA	NA	NA	0.00000	NA	NA	NA	NA	NA
15573768	c.1954 − 428A > C	NA	rs2873356	3.90	NA	NA	NA	P	NA	0.2257	NA	0.31059	NA	NA
15586448	c.1897 − 499T > G	NA	rs1514280	3.93	NA	NA	NA	P	NA	0.1979	NA	0.28068	NA	NA
15585933	c.1913A > G	N638S	rs183135788	22.5	NA	NA	NA	DC	0.00029	0.0005	NA	0.00018	NA	NA
15543160	c.753 − 251A > C	NA	rs4830965	7.27	NA	NA	NA	P	NA	0.1756	NA	0.28718	NA	NA
15540299	c.511 − 170T > C	NA	rs1476524	8.0	NA	NA	NA	P	NA	0.3105	NA	0.38494	NA	NA
15593829	c.1402A > G	I468V	rs191860450	26.1	DE	POD	NE	DC	0.00073	0.0005	NA	0.00064	NA	NA
15618884	c.151A > G	N51D	rs760159085	25.1	DE	POD	DE	DC	0.00002	NA	NA	NA	NA	NA
15613063	c.250C > A	P84T	rs759134032	2.85	DE	Benign	NE	P	0.00001	NA	NA	NA	NA	NA
15603630	c.868A > C	N290H	rs763994205	1.10	DE	PRD	DE	P	0.00001	NA	NA	NA	NA	NA
15572312	c.1553G > C	R518T	rs1158307424	28.5	DE	PRD	NE	P	NA	NA	NA	NA	NA	NA
15591500	c.1531T > C	S511P	NA	26.9	DE	POD	NE	DC	NA	NA	NA	NA	NA	NA
15599378	c.1036C > T	P346S	rs1410274315	22.4	DE	PRD	NE	DC	NA	NA	NA	NA	NA	NA
15591521	c.1510T > A	F504I	rs760281053	25.9	DE	PRD	DE	P	0.00001	NA	NA	NA	NA	NA
15596412	c.1097T > C	M366T	rs758568640	25.0	DE	PRD	DE	DC	0.00001	NA	NA	NA	NA	NA
15593893	c.1338T > G	I446M	rs1290769028	11.74	DE	Benign	NE	DC	NA	NA	NA	0.00005	NA	NA
15618973	c.62T > C	I21T	rs1244687367	0.24	DE	Benign	NE	P	NA	NA	NA	NA	NA	NA
15596388	c.1121A > G	H374R	rs1309363592	25.1	DE	PRD	DE	DC	NA	NA	NA	NA	NA	NA
15596317	c.1192G > A	E398K	rs772619843	37	DE	PRD	NE	DC	0.00001	NA	NA	NA	NA	NA
15591503	c.1528T > C	Y510H	rs779199005	29.2	DE	PRD	DE	DC	NA	0.0003	NA	NA	NA	NA
15618917	c.118T > C	F40L	NA	9.11	DE	Benign	NE	P	NA	NA	NA	NA	NA	NA
15618959	c.76A > G	K26E	rs1299103394	9.61	DE	Benign	NE	P	NA	NA	NA	NA	NA	NA
15618930	c.105A > C	E35D	NA	3.56	NA	Benign	NE	DC	NA	NA	NA	NA	NA	NA
15589833	c.1750_1751delCTinsGC	L584A	NA	NA	DE	NA	NA	NA	NA	NA	NA	NA	NA	NA
15596297	c.1210_1212delGTTinsAAA	V404K	NA	NA	DE	NA	NA	NA	NA	NA	NA	NA	NA	NA
15618063	c.186 + 786A > T	NA	rs1978124	0.32	NA	NA	NA	P	NA	0.2053	NA	0.37498	NA	NA
15583904	c.2114 + 472G > T	NA	rs714205	9.18	NA	NA	NA	P	NA	0.3083	NA	0.18440	NA	NA
15597509	c.1071 − 1071G > A	NA	rs4646155	5.40	NA	NA	NA	P	NA	0.0615	NA	0.03570	NA	NA
15584488	c.2002G > A	E668K	rs200180615	35	DE	Benign	NE	DC	0.00002	0.0005	NA	NA	NA	NA
15585879	c.1967T > G	L656T	rs199951323	1.05	DE	POD	NE	P	0.00001	0.0003	NA	NA	NA	NA
15586964	c.1897 − 1015G > C	NA	rs4240157	5.54	NA	NA	NA	P	NA	0.3179	NA	0.38257	NA	NA
15618061	c.186 + 788T > G	NA	rs2106809	3.14	NA	NA	NA	P	NA	0.3163	NA	0.19141	NA	NA
15582966	c.2115 − 625C > T	NA	rs233575	0.99	NA	NA	NA	P	NA	0.1367	NA	0.22801	NA	NA
15591662	c.1443 − 74G > A	NA	rs4646192	0.80	NA	NA	NA	P	NA	0.0019	NA	0.00312	NA	NA
15607588	c.584 − 8delA	NA	rs752472046	NA	NA	NA	NA	NA	NA	NA	NA	NA	NA	NA
15579969	c.^*∗*^59G > A	NA	NA	2.06	NA	NA	NA	P	NA	NA	NA	NA	NA	NA
15605942	c.736G > A	A246T	NA	0.08	DE	Benign	NE	P	NA	NA	NA	NA	NA	NA
15607532	c.631G > A	G211R	rs148771870	12.86	DE	POD	NE	P	0.00140	NA	NA	0.00091	NA	NA
15591530	c.1501G > A	A501T	rs140473595	24.9	DE	POD	NE	DC	0.00001	0.0003	NA	0.00009	NA	NA
15591517	c.1514A > G	H505R	rs1016409802	26.6	DE	PRD	DE	DC	NA	NA	NA	NA	NA	NA
15591539	c.1492T > C	C498R	NA	27.1	DE	PRD	DE	DC	NA	NA	NA	NA	NA	NA
15599350	c.1064A > C	D355A	NA	24.7	DE	PRD	DE	DC	NA	NA	NA	NA	NA	NA
15609932	c.487T > A	W163R	NA	24.8	DE	POD	DE	P	NA	NA	NA	NA	NA	NA
15596295	c.1214G > A	G405E	NA	26.0	DE	PRD	DE	DC	NA	NA	NA	NA	NA	NA
15605924	c.754T > A	Y252N	NA	24.6	DE	PRD	DE	P	NA	NA	NA	NA	NA	NA
15593863	c.1368A > C	L456F	NA	10.53	DE	PRD	DE	DC	NA	NA	NA	NA	NA	NA
15607537	c.626T > G	V209G	NA	0.41	DE	Benign	NE	P	NA	NA	NA	NA	NA	NA
15596379	c.1130G > A	G377E	rs767462182	26.8	DE	PRD	DE	DC	0.00001	NA	NA	NA	NA	NA
15607493	c.670G > A	E224K	NA	33	DE	Benign	NE	DC	NA	NA	NA	NA	NA	NA
15589846	c.1738A > G	N580D	NA	15.16	DE	Benign	NE	P	NA	NA	NA	NA	NA	NA
15605887	c.791C > G	A264G	NA	27	DE	POD	DE	P	NA	NA	NA	NA	NA	NA
15609885	c.533_534delCAinsAC	P178H	NA	NA	DE	NA	NA	NA	NA	NA	NA	NA	NA	NA
15609928	c.490_491delGCinsCT	A164L	NA	NA	DE	NA	NA	NA	NA	NA	NA	NA	NA	NA
15610405	c.385_386delACinsCT	T129L	NA	NA	DE	NA	NA	NA	NA	NA	NA	NA	NA	NA
15612979	c.334A > G	K112E	NA	19.94	DE	Benign	NE	P	NA	NA	NA	NA	NA	NA
15613119	c.194C > T	A65V	NA	19.33	DE	Benign	NE	P	NA	NA	NA	NA	NA	NA
15613038	c.275C > T	T92I	rs763395248	1.42	DE	Benign	DE	P	0.00002	NA	NA	NA	NA	NA
15618872	c.163A > G	T55A	rs775273812	24.4	DE	Benign	DE	DC	0.00001	NA	NA	NA	NA	NA
15619013	c.22C > T	L8F	rs201035388	12.24	DE	Benign	NE	p	0.00007	NA	NA	NA	NA	NA
15591514	c.1517T > C	V506A	rs775181355	27.1	DE	PRD	DE	DC	0.00001	NA	NA	NA	NA	NA
15596343	c.1166C > A	P389H	rs762890235	24.5	DE	PRD	DE	DC	0.00003	NA	NA	NA	NA	NA
15566355	c.2012G > C	R671P	rs753705431	11.18	DE	Benign	NE	P	0.00001	NA	NA	NA	NA	NA
15618942	c.93G > A	K31=	rs758278442	0.00	DE	Benign	NE	P	0.00002	NA	NA	NA	NA	NA
15582790	c.2115 − 449G > A	NA	rs2074192	3.02	NA	NA	NA	P	NA	0.3632	NA	0.42428	NA	NA
15589028	c.1838 − 552A > G	NA	rs4646171	2.89	NA	NA	NA	P	NA	0.0702	NA	0.04424	NA	NA
15572684	c.1542 − 361G > C	NA	rs879922	0.94	NA	NA	NA	P	NA	0.3176	NA	0.38084	NA	NA
15582747	c.2115 − 406A > G	NA	rs1514283	0.39	NA	NA	NA	P	NA	0.1094	NA	0.08324	NA	NA
15569381	c.1896 + 914G > C	NA	rs4646176	2.13	NA	NA	NA	P	NA	0.0694	NA	0.04411	NA	NA
15601343	c.901 − 1830T > C	NA	rs4646188	6.19	NA	NA	NA	P	NA	0.0437	NA	0.10405	NA	NA
15558483	g.62707C > A	NA	rs4830542	NA	NA	NA	NA	P	NA	0.3158	NA	0.37655	NA	NA
15608386	c.584 − 807G > A	NA	rs2158083	0.44	NA	NA	NA	P	NA	0.1918	NA	0.29656	NA	NA
15618896	c.139T > C	S47P	NA	21.7	DE	PRD	DE	P	NA	NA	NA	NA	NA	NA
15618863	c.172A > C	N58H	rs1222417695	24.4	DE	PRD	DE	DC	NA	NA	NA	NA	NA	NA
15612970	c.343C > T	R115W	rs1292756480	9.33	DE	PRD	DE	P	NA	NA	NA	NA	NA	NA
15610369	c.421_422delTGinsAC	C141T	rs1222417695	NA	DE	POD	DE	NA	NA	NA	NA	NA	NA	NA
15609868	c.551T > C	V184A	rs75814285	28.8	DE	PRD	DE	DC	NA	NA	NA	NA	NA	NA
15609848	c.571G > C	A191P	rs765733397	27.2	DE	PRD	DE	DC	0.00001	NA	NA	NA	NA	NA
15607513	c.650A > G	Y217C	rs1300152093	23.3	DE	PRD	DE	DC	NA	NA	NA	NA	NA	NA
15607507	c.656G > A	R219H	rs759590772	23.4	DE	PRD	NE	DC	0.00009	NA	NA	NA	NA	NA
15587851	c.704C > G	P235R	rs1172580854	25.8	DE	PRD	DE	P	NA	NA	NA	NA	NA	NA
15605923	c.755A > G	Y252C	rs771769548	24.5	DE	PRD	DE	DC	0.00002	NA	NA	NA	NA	NA
15605891	c.787C > T	P263S	rs200745906	25.3	DE	PRD	DE	DC	0.00005	NA	NA	0.00005	NA	NA
15603690	c.808A > G	M270V	rs766319182	25.4	DE	PRD	DE	DC	0.00001	NA	NA	NA	NA	NA
15603651	c.847G > T	V283F	rs1203006090	18.56	DE	PRD	DE	P	NA	NA	NA	NA	NA	NA
15603635	c.863A > C	K288T	NA	23.1	DE	PRD	DE	P	NA	NA	NA	NA	NA	NA
15585503	c.872T > A	I291K	rs756358940	26.6	DE	PRD	DE	P	0.00003	NA	NA	NA	NA	NA
15603623	c.875A > T	D292V	NA	29.3	DE	PRD	DE	P	NA	NA	NA	NA	NA	NA
15607528	c.634_635delGTinsAA	V212K	NA	NA	DE	Benign	NE	NA	NA	NA	NA	NA	NA	NA
15596384	c.1125G > C	E375D	NA	25.6	DE	PRD	DE	P	NA	NA	NA	NA	NA	NA
15596320	c.1189A > G	N397D	rs1365935088	25.8	DE	PRD	DE	DC	NA	NA	NA	0.00005	NA	NA
15596311	c.1198T > C	F400L	rs1214851578	27.1	DE	PRD	DE	DC	NA	NA	NA	NA	NA	NA
15596281	c.1228C > G	L410V	NA	25.8	DE	PRD	DE	P	NA	NA	NA	NA	NA	NA
15596256	c.1253T > C	L418S	rs1466701781	35	DE	PRD	DE	DC	NA	NA	NA	NA	NA	NA
15596250	c.1259C > G	S420C	NA	22.2	DE	PRD	DE	P	NA	NA	NA	NA	NA	NA
15596230	c.1279G > T	D427Y	rs1316056737	15.34	DE	PRD	DE	P	NA	NA	NA	NA	NA	NA
15593921	c.1310A > G	N437S	NA	26.9	DE	PRD	DE	P	NA	NA	NA	NA	NA	NA
15593897	c.1334C > T	T445M	rs764772589	21.9	DE	PRD	DE	DC	0.00001	NA	NA	NA	NA	NA
15593892	c.1339G > T	V447F	rs776328956	17.53	DE	PRD	DE	DC	0.00007	NA	NA	0.00014	NA	NA
15593888	c.1343G > A	G448E	rs763655186	25.0	DE	PRD	DE	DC	0.00001	NA	NA	NA	NA	NA
15593845	c.1386G > A	M462I	rs1463563888	23.8	DE	PRD	NE	DC	NA	NA	NA	NA	NA	NA
15591586	c.1445G > A	R482Q	rs748359955	26.0	DE	PRD	NE	DC	0.00002	NA	NA	0.00005	NA	NA
15591491	c.1540C > G	R514G	rs1352194082	27.7	DE	PRD	DE	P	NA	NA	NA	NA	NA	NA
15590421	c.1567T > C	F523L	NA	27.4	DE	PRD	DE	DC	NA	NA	NA	NA	NA	NA
15572243	c.1622A > T	K541I	rs889263894	23.9	DE	PRD	DE	P	NA	NA	NA	0.00005	NA	NA
15589875	c.1709T > C	L570S	rs1305384714	24.7	DE	PRD	DE	DC	NA	NA	NA	0.00005	NA	NA
15585885	c.1961A > C	Y654S	rs1479485636	23.3	DE	PRD	DE	DC	NA	NA	NA	0.00005	NA	NA
15584404	c.2086C > A	P696T	rs755445931	23.3	DE	PRD	DE	DC	0.00001	NA	NA	NA	NA	NA
15584392	c.2098G > A	V700I	rs1392981937	25.8	DE	PRD	NE	DC	NA	NA	NA	NA	NA	NA
15582334	c.2122C > T	R708W	rs776995986	2.26	DE	PRD	DE	DC	0.00001	NA	NA	0.00009	NA	NA
15582328	c.2128C > T	R710C	rs901495523	25.5	DE	PRD	DE	DC	NA	NA	NA	0.00009	NA	NA
15582291	c.2165T > C	L722P	NA	24.6	DE	PRD	NE	DC	NA	NA	NA	NA	NA	NA
15580092	c.2353_2354delGAinsAC	D785T	NA	NA	DE	POD	NE	NA	NA	NA	NA	NA	NA	NA
15580035	c.2411C > T	S804F	rs771107251	23.9	DE	PRD	NE	DC	0.00001	NA	NA	NA	NA	NA

The table reports the genomic position, the nucleotide, and amino acid change of identified variants in the ACE2 gene. These data are based on the Genome Reference Consortium Human Build 37 (GRCh37). ^1^CADD, Phred ≤20: neutral; Phred >20: damaging; ^2^SIFT, score ≤0.05: deleterious; score >0.05: tolerable; ^3^polyphen-2, score = 0–0.15: benign; score = 0.15–0.85: possibly damaging; score = 0.85–1: probably damaging; ^4^PROVEAN, score ≤ −2.5: deleterious; score > −2.5: neutral; TO: tolerable; DE: deleterious; NE: natural, DC: disease causing; NA: not available. PRD: probably damaging; POD: possibly damaging; P: polymorphism.

**Table 2:** The most pathogenic variants of the *ACE*2 gene.

Position on chromosome X	Nucleotide change	Amino acid change	dbSNP	CADD Phred	SIFT	Polyphen2	PROVEAN	Mutation taster	ExAc	1000 genomes project	ESP6500	gnomAD	Iranome	GME
15618926	c.109G > A	E37K	rs146676783	34	DE	POD	NE	DC	0.00002	NA	0.0284	0.00009	NA	NA
15591503	c.1528T > C	Y510H	rs779199005	29.2	DE	PRD	DE	DC	NA	0.0003	NA	NA	NA	NA
15609868	c.551T > C	V184A	rs75814285	28.8	DE	PRD	DE	DC	NA	NA	NA	NA	NA	NA
15596380	c.1129G > T	G377Q	NA	28.2	DE	PRD	DE	DC	NA	NA	NA	NA	NA	NA
15596361	c.1148T > C	M383T	rs1396769231	27.7	DE	PRD	DE	DC	NA	NA	NA	NA	NA	NA

The table reports the genomic position, the nucleotide, and amino acid change of the most pathogenic variants in the *ACE2* gene. ^1^CADD, Phred ≤20: neutral; Phred >20: damaging; ^2^SIFT, score ≤0.05: deleterious; score >0.05: tolerable; ^3^polyphen-2, score = 0–0.15: benign; score = 0.15–0.85: possibly damaging; score = 0.85–1: probably damaging; ^4^PROVEAN, score ≤ −2.5: deleterious; score > −2.5: neutral; TO: tolerable; DE: deleterious; NE: natural, DC: disease causing; NA: not available. PRD: probably damaging; POD: possibly damaging; P: polymorphism.

## Data Availability

All the data generated or analyzed during this study are included in this published article. The datasets generated and/or analyzed during the current study are available in the HGMD (https://www.hgmd.cf.ac.uk/ac/index.php), ClinVar (https://www.ncbi.nlm.nih.gov/clinvar/), and Google Scholar (https://scholar.google.com/).
